# Exploring the impact of acute viral exposure on clinical characteristics and antibody profiles in antiphospholipid syndrome: a study in CAPSTONE

**DOI:** 10.1007/s10238-024-01400-5

**Published:** 2024-06-18

**Authors:** Chuhan Wang, Hui Jiang, Siyun Chen, Yuan Zhao, Jun Li, Can Huang, Yangzhong Zhou, Qian Wang, Xinping Tian, Mengtao Li, Xiaofeng Zeng, Yan Zhao, Chuancong Wu, Jiuliang Zhao

**Affiliations:** 1 Department of Rheumatology and Clinical Immunology, Peking Union Medical College Hospital, Beijing, 100730 China; 2 National Clinical Research Center of Dermatologic and Immunologic Diseases (NCRC-DID), Beijing, 100730 China; 3 Key Laboratory of Rheumatology and Clinical Immunology, Ministry of Education, Beijing, 100730 China; 4 State Key Laboratory of Complex Severe and Rare Diseases, Peking Union Medical College Hospital, Beijing, 100730 China; 5 Department of Rheumatology and Immunology, Affiliated Qingyuan Hospital, The Sixth Clinical Medical School, Guangzhou Medical University, Qingyuan People’s Hospital, Qingyuan, 511518 Guangdong China

**Keywords:** SARS-CoV-2, Antiphospholipid syndrome, Antiphospholipid antibody, Thrombocytopenia

## Abstract

The relationship between antiphospholipid syndrome (APS) and acute viral infection, such as SARS-CoV-2, is unclear. This study aims to assess symptoms, antiphospholipid antibody (aPL) fluctuations, and complication risks in APS patients infected with SARS-CoV-2. APS patients from Peking Union Medical College Hospital during the COVID-19 outbreak (October–December 2022) were included. Age- and gender-matched APS patients without infection served as controls. Data on demographics, symptoms, treatments, and serum aPL levels were analyzed. Of 234 APS patients, 107 (45.7%) were infected with SARS-CoV-2. Typical symptoms included high fever (81.3%), cough/expectoration (70.1%), and pharyngalgia (52.3%). Age- and gender-based matching selected 97 patients in either infected or uninfected group. After infection, anti-β-2-glycoprotein I-IgG (aβ2GP1-IgG) increased from 4.14 to 4.18 AU/ml, aβ2GP1-IgM decreased from 9.85 to 7.38 AU/ml, and anticardiolipin-IgA (aCL-IgA) significantly increased with a median remaining at 2.50 APLU/ml. Lupus anticoagulants and other aPLs remained stable. Arterial thrombosis incidence increased from 18 (18.6%) to 21 (21.6%), while venous thrombosis incidence did not change. Additionally, 7 (6.5%) patients presented either new-onset or worsening thrombocytopenia, characterized by a significant decline in platelet count (no less than 10 × 10^9^/L) within two weeks of SARS-CoV-2 infection, all of which recovered within 2 weeks. Acute SARS-CoV-2 infection may induce or worsen thrombocytopenia but does not substantially increase thrombotic events in APS. The process of SARS-CoV-2 infection was related to mild titer fluctuation of aβ2GP1-IgG, aβ2GP1-IgM and aCL-IgA in APS patients, necessitating careful monitoring and management.

## Background

Antiphospholipid syndrome (APS) is a rare autoimmune disease of unknown etiology characterized by thromboembolism, obstetric morbidity, and extra-criteria microvascular manifestations with persistent elevation of pathogenic antiphospholipid antibodies (aPLs) [1]. Viral infection has been considered one of the major environmental factors triggering autoimmune disease [2, 3]. Multiple mechanisms including molecular mimicry, bystander activation, and epitope spreading lead to the breakdown of self-tolerance caused by viral infection [4–6]. In APS, viral infection has been reported to be related to both the production of aPLs and changing clinical manifestations. Elevated anticardiolipin antibody (aCL) is found in patients with APS infected with hepatitis C virus (HCV) [7, 8]. Lupus anticoagulant (LA) and aCL positivity were reported in those infected with human immunodeficiency virus (HIV) [9, 10]. Sporadic cases of other viral infections, including Epstein–Barr virus (EBV), varicella virus, and cytomegalovirus (CMV), have also been reported to cause elevation of aPL levels [11–18]. The co-occurrence of APS and viral infections may lead to additional comorbidities such as valve vegetation [19]. However, most research are based on cross-sectional studies of the relationship between aPLs and viral infection, with very limited detection of the effect of acute viral outbreaks in APS cohorts. Recently, the entire world has been threatened by emerging new diseases, including severe acute respiratory syndrome (SARS), avian influenza, and coronavirus disease 19 (COVID-19) [20–22]. Considering the effect of viral infection on APS and the unpredictability of viral outbreaks, it is necessary to build a model to detect the effects of acute viral pandemic outbreaks in patients with APS.

Since its emergence in December 2019, the SARS-CoV-2 population has experienced several waves. Following the relaxation of lockdown measures in China, there was a significant peak in infections from October 2022 to January 2023, providing a crucial opportunity to study the short-term impact of a viral explosion on the APS cohort. Within our regular follow-up APS cohort, 107 patients infected SARS-CoV-2. We use this phase as a relevant model to assess the impact of the viral outbreak on APS. Here, we present the alterations in APS manifestations, aPL titers, and laboratory indices over the three months following infection, aiming to elucidate the specific impacts of viral outbreaks on APS.

## Methods

### Subjects and matching

**Fig. 1:**
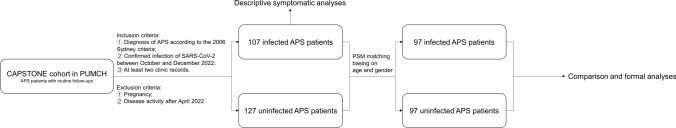
Schematic diagram of the study workflow

Patients diagnosed with APS who had routine follow-ups at Peking Union Medical College Hospital were considered for the study. The inclusion criteria included: (i) diagnosis of APS according to the 2006 Sydney APS classification criteria, (ii) confirmed infection of SARS-CoV-2 between October and December 2022, and (iii) at least two records of visiting the clinic with two sets of laboratory test results (one between October and November 2022 and the other between January and March 2023). Exclusion criteria were: (i) pregnancy or (ii) disease activity after April 2022 defined as having newly developed thrombosis, obstetric events or clinical manifestations requiring immunosuppressive therapy. Initially, 107 infected APS patients and 127 uninfected APS patients were included. An age- and gender-based 1:1 matching was conducted using the propensity score matching (PSM) method, after which 97 patients in both groups were finally included (Fig. 1).

### Collection of clinical data

Baseline clinical characteristics of APS patients were collected between October and November 2022 from the latest visit before SARS-CoV-2 infection if the patient was infected. These included age, gender, age of APS onset, age of APS diagnosis, duration before diagnosis, underlying diseases, APS-related manifestations, and aPL titers. Viral infection-related clinical symptoms and severity were recorded during the SARS-CoV-2 infection. Newly developed clinical manifestations including thrombosis, thrombocytopenia, microvascular diseases, laboratory tests, and aPL titers change were collected between January and March 2023 from the visits after SARS-CoV-2 infection.

### Laboratory tests

Serum aPLs included aCL, anti-β2 glycoprotein 1 (aβ2GP1) antibodies and LA. LA was measured according to recommendation by the Scientific and Standardization Committee [23]. To be specific, LA was measured using activated partial thromboplastin time-based assay (aPTT) and the dilute Russell viper venom time (dRVVT), and positivity was defined as an aPTT ratio greater than 1.20 or a dRVVT ratio over than 1.20. The aCL and aβ2GP1 were measured by chemiluminescent immunoassay (CLIA) (iFlash CLIA kits provided by YHLO Biotech Co., Shenzhen, China). According to the manufacture's instruction, medium or high titer of aCL was defined by a cutoff value of 10U/mL and medium or high titer of aβ2GP1 was defined by a cutoff value of 20U/mL. This detection system showed good sensitivity and specificity in our cohort in the previous study [24].

### Statistical analyses

Measurements were presented as median (Quartiles). Intragroup comparisons were analyzed mainly by Wilcoxon–Mann–Whitney test as none of the parameters fit in normal distribution. Counts were presented as rates. Intragroup comparisons were analyzed by **χ**^2^ test or Fisher’s exact test as appropriate. For self-comparison, matched Wilcoxon–Mann–Whitney test and McNemar’s test were applied. Age- and gender- matched SARS-CoV-2 uninfected patients were selected by propensity score matching (PSM) with a 1:1 ratio. All statistical analyses were performed using IBM SPSS Statistics 25.0 and R studio software, with *p* < 0.05 considered statistically significant.

## Results

### Baseline characteristics of the matched APS cohort

For all 107 infected patients and 127 uninfected patients, demographic characteristics, APS manifestations, aPL positivity and titers, vaccination status, and basic therapy for APS at baseline were collected and displayed (Table 1). Ninety-seven patients in each group fit in the cohort matched by age and gender. The baseline characteristics of the matched cohort are also shown in Table 1. It can be observed that the significant differences between the unmatched groups of gender, arterial thrombotic events and thrombocytopenia were eliminated in the comparison of the matched groups. At baseline, patients with SARS-CoV-2 infection had a median age of 34 years, which leveled with the median age of 35 years of patients without infection. Patients with primary APS (PAPS) were more common in the infected group (73.2%) compared to the uninfected group (57.7%) (*p* = 0.024). There was no significant difference between the two groups in clinical manifestations regarding thrombotic events, thrombocytopenia and valvular heard disease. Double positivity was lower in the infected group (17.5%) compared to the uninfected group (29.9%) (*p* = 0.043). A lower proportion of vaccinated individuals were found in the infected group (42.3%) compared to the uninfected group (56.7%) (*p* = 0.044). As for treatment, the use of antiplatelet and anticoagulant therapies, including low molecular weight heparin (LMWH), warfarin, direct oral anticoagulants (DOACs), and combined antiplatelet and anticoagulant therapy, showed no significant differences between the groups. Roughly one third of patients received glucocorticoid therapy, and most received hydroxychloroquine (79.4%). Conventional synthetic disease-modifying antirheumatic drugs (csDMARDS) were used by approximately 1/3 of patients, while only a small percentage of patients received biologic/targeted synthetic disease-modifying antirheumatic drugs (b/tsDMARDS). In those treated with glucocorticoid, most patients were secondary to SLE, and other PAPS patients had thrombocytopenia before.

**Table 1:** Baseline characteristics of total and matched APS patients with/without COVID-19 infection

	Pre-PSM			Post-PSM		
	Patients in the infected subgroup (*N* = 107)	Patients in the uninfected subgroup (*N* = 127)	*p* value	Patients in the infected subgroup (*N* = 97)	Patients in the uninfected subgroup (*N* = 97)	*p* value
Age (years)	34 [30, 42]	35[32, 44]	0.355	34 [30,42]	35 [31,44]	0.480
Gender (female, *n* (%))	79 (73.8%)	108 (85.0%)	***0.033***	79 (81.4%)	78 (80.4%)	0.855
PAPS, * n* (%)	79 (73.8%)	78 (61.4%)	***0.044***	71 (73.2%)	56 (57.7%)	***0.024***
Clinical manifestations						
Venous thrombotic events, * n* (%)	37 (34.6%)	31 (24.4%)	0.088	32 (33.0%)	27 (27.8%)	0.435
Arterial thrombotic events, * n* (%)	20 (18.7%)	12 (9.4%)	***0.040***	18 (18.6%)	11 (11.3%)	0.159
Thrombocytopenia, * n* (%)	37(34.6%)	29 (22.8%)	***0.047***	29 (29.9%)	21 (21.6%)	0.189
Valvular heart disease, * n* (%)	3 (2.8%)	9 (7.1%)	0.139	3 (3.1%)	6 (6.2%)	0.306
Antibody profiles						
aCL-IgG/M, * n* (%)	51 (47.7%)	70 (55.1%)	0.256	43 (44.3%)	50 (51.5%)	0.314
aβ2GP1-IgG/M, * n* (%)	75 (70.1%)	91 (71.7%)	0.793	67 (69.1%)	68 (70.1%)	0.876
LA, * n* (%)	54 (50.5%)	64 (50.4%)	0.991	44 (45.4%)	48 (49.5%)	0.565
Single positivity, * n* (%)	41 (38.3%)	33 (26.0%)	***0.043***	39 (40.2%)	27 (27.8%)	0.069
Double positivity, * n* (%)	17 (15.9%)	36 (28.3%)	***0.023***	17 (17.5%)	29 (29.9%)	***0.043***
Triple positivity, * n* (%)	35 (32.7%)	40 (31.5%)	0.843	27 (27.8%)	27 (27.8%)	1.000
Vaccination (vaccinated, * n* (%))	44 (41.1%)	66 (52.0%)	0.098	41 (42.3%)	55 (56.7%)	***0.044***
Treatment						
Antiplatelet, * n* (%)	68 (63.6%)	78 (61.4%)	0.737	61 (62.9%)	59 (60.8%)	0.768
Anticoagulant, * n* (%)	51 (47.7%)	58 (45.7%)	0.761	45 (46.4%)	50 (51.5%)	0.473
LMWH, * n* (%)	11 (10.3%)	17 (13.4%)	0.466	11 (11.3%)	14 (14.4%)	0.520
Warfarin, * n* (%)	30 (28.0%)	23 (18.1%)	0.071	26 (26.8%)	19 (19.6%)	0.234
DOACs, * n* (%)	12 (11.2%)	13 (10.2%)	0.809	10 (10.3%)	12 (12.4%)	0.651
Antiplatelet & anticoagulant, * n* (%)	24 (22.4%)	24 (18.9%)	0.505	21 (21.6%)	22 (22.7%)	0.863
Glucocorticoids						
Low dose, * n* (%)	23 (21.5%)	31 (24.4%)	0.598	23 (23.7%)	27 (27.8%)	0.511
High dose, * n* (%)	5 (4.7%)	12 (9.4%)	0.161	3 (3.1%)	9 (9.3%)	0.074
Hydroxychloroquine, * n* (%)	85 (79.4%)	101 (79.5%)	0.987	77 (79.4%)	81 (83.5%)	0.460
cs DMARDS, * n* (%)	31 (29.0%)	42 (33.1%)	0.500	26 (26.8%)	33 (34.0%)	0.275
b/t DMARDS, * n* (%)	1 (0.9%)	5 (3.9%)	0.148	1 (0.9%)	3 (2.8%)	0.307
Laboratory indexes						
WBC (× 10^9^/L)	6.24 [4.53, 7.91]	6.28 [5.07, 8.10]	0.307	6.20 [4.47,7.55]	6.50 [5.09, 8.01]	0.284
HGB (g/L)	132.00 [120.00, 144.00]	131.00 [120.00, 137.00]	0.236	132.00 [120.00,141.00]	132.00 [121.00, 138.50]	0.871
PLT (× 10^9^/L)	214.00 [150.00, 263.00]	213.00 [156.00, 271.00]	0.726	215.00 [157.00,263.00]	217.00 [156.00, 271.00]	0.664
IgG (g/L) (Normal range: 7–17)	11.36 [9.56, 13.65]	11.19 [9.56, 13.04]	0.605	11.40 [9.68,13.69]	11.47 [9.60, 13.12]	0.739
IgM (g/L) (Normal range: 0.4–2.3)	1.29 [0.88, 1.79]	1.14 [0.74, 1.61]	0.072	1.30 [0.88 1.78]	1.13 [0.72, 1.52]	0.055
IgA (g/L) (Normal range: 0.7–4)	1.88 [1.44, 2.47]	2.20 [1.59, 2.83]	***0.024***	1.89 [1.44 2.49]	2.22 [1.62, 2.96]	***0.016***
Antibody titers						
aCL						
IgG (GPLU/ml) (Normal range: 0–8)	4.74 [2.16, 28.70]	4.01 [2.09, 38.50]	0.765	4.45 [2.16, 29.50]	4.01 [2.10, 37.05]	0.809
IgM (MPLU/ml) (Normal range: 0–8)	4.04 [2.00, 8.87]	4.11 [2.00, 11.1]	0.464	4.04 [2.00, 8.78]	3.64 [0.00, 8.80]	0.770
IgA (APLU/ml) (Normal range: 0–8)	2.50 [2.50, 3.96]	2.50 [2.50, 8.15]	***0.000***	2.50 [2.50, 3.41]	2.57 [0.00, 8.26]	***0.000***
aβ2GPI (AU/ml)						
IgG (Normal range: 0–16)	5.63 [2.16, 28.30]	3.72 [2.22, 45.2]	0.681	4.14 [2.16, 25.85]	3.67 [2.22, 40.45]	0.567
IgM (Normal range: 0–16)	9.15 [2.00, 38.70]	9.66 [2.00, 44.30]	0.588	9.85 [2.00, 36.2]	4.97 [0.00, 37.00]	0.849
IgA (Normal range: 0–16)	2.11 [2.50, 5.27]	3.00 [2.50, 10.60]	0.086	2.50 [2.50, 4.60]	3.05 [0.00 11.70]	0.053
LA	1.20 [1.03, 1.51]	1.20 [1.20, 1.44]	0.552	1.16 [1.03, 1.44]	1.16 [1.03 1.42]	0.997

*p* value lower than 0.05 is presented in bolditalics

LMWH: Low molecular weight heparin; DOACs: Direct oral anticoagulants; DMARDS: Disease-modifying antirheumatic drugs

### Overall description of the clinical manifestations of SARS-CoV-2 infection in APS patients

Clinical manifestations in patients with APS who were infected by SARS-CoV-19 were firstly documented (Table 2). Previous comprehensive meta-analyses describing symptoms of healthy Chinese individuals with SARS-CoV-19 infection were included and briefly compared. In our general APS cohort with 107 patients, the majority exhibited a fever higher than 38 °C, accompanied by shivering/myalgia and cough/expectoration. Throughout the study, only a few patients (2.8%) experienced severe complications leading to hospitalization. In total, three patients suffered from pneumonia, one experienced carditis. In total, three patients suffered from pneumonia, one experienced carditis, three had new-onset thromboembolic events, and seven developed new-onset thrombocytopenia or experienced a platelet descent of no less than 10 × 10^9^/L under a thrombocytopenic background. The incidences of clinical symptoms such as cough / expectoration, pharyngalgia, shiver/myalgia and headache were higher in the infected group compared to the normal population.

**Table 2:** Epidemiology of COVID-19 infection in 107 APS patients and normal population derived from previous work

	Infected APS patients (*N* = 107)	Infected normal population derived from meta-analyses
Fever (≤ 38℃), *n* (%)	20 (18.7%)	22.0% [25]
Fever (> 38℃), *n* (%)	87 (81.3%)	78.0% [25]
Duration (days)	2 [1, 3]	NA
Shiver/myalgia, *n* (%)	52 (48.6%)	15.2%/21.9% [26]
Headache, *n* (%)	25 (23.4%)	11.3% [26]
Pharyngalgia, *n* (%)	56 (52.3%)	11.6% [26]
Cough/expectoration, *n* (%)	75 (70.1%)	58.3%/23.7% [26]
Chest pain/tightness, *n* (%)	6 (5.6%)	22.9%[26]
Smell/taste loss, *n* (%)	11 (10.3%)	NA
Fatigue, *n* (%)	28 (26.2%)	34.0%[26]
Diarrhea/vomit, *n* (%)	8 (7.5%)	8.2%[26]
Pneumonia, *n* (%)	3 (2.8%)	NA
Myocarditis, *n* (%)	1 (0.9%)	NA
Newly-onset thrombotic events, *n* (%)	3 (2.8%)	NA
Newly-onset obstetric morbidity, *n* (%)	0 (0%)	NA
Newly-onset thrombocytopenia, *n* (%)	7 (6.5%)	NA
Hospitalization, *n* (%)	3 (2.8%)	NA
Death, *n* (%)	0 (0%)	NA

### Characteristics of the matched cohort after COVID-19 outbreak

The clinical manifestations and laboratory indices of the 97 matched APS patients post-SARS-CoV-2 infection compared to those who were not infected were presented (Table 3). There was no significant difference in the occurrence of venous thrombotic events between the infected group (32, 33.0%) and the uninfected group (28, 28.9%) (*p* = 0.534). The infected group exhibited a higher occurrence of arterial thrombotic events (21, 21.6%) compared to the uninfected group (11, 11.3%) approaching statistical significance (*p* = 0.053), and thrombocytopenia was also significantly more prevalent in the infected group (35, 36.1%) compared to the uninfected group (22, 21.6%) (*p* = 0.040). Regarding to laboratory tests, the median WBC count was significantly lower in the infected group (*p* = 0.009), while the median IgM level was significantly higher in the infected group (*p* = 0.049). Both aCL-IgA titers and aβ2GP1-IgA titers were significantly higher in the infected group (*p* < 0.001 and *p* = 0.045, respectively).

**Table 3:** Characteristics of total and matched APS patients after COVID-19 outbreak

	Pre-PSM			Post-PSM		
	Patients in the infected subgroup (*N* = 107)	Patients in the uninfected subgroup (*N* = 127)	*p* value	Patients in the infected subgroup (*N* = 97)	Patients in the uninfected subgroup (*N* = 97)	*p* value
Clinical manifestations						
Venous thrombotic events, *n* (%)	37 (34.6%)	32 (25.2%)	0.117	32 (33.0%)	28 (28.9%)	0.534
Arterial thrombotic events, *n* (%)	23 (21.5%)	12 (9.4%)	***0.010***	21 (21.6%)	11 (11.3%)	0.053
Thrombocytopenia, *n* (%)	44 (41.1%)	31 (24.4%)	***0.006***	35 (36.1%)	22 (21.6%)	***0.040***
Laboratory indexes						
WBC (× 10^9^/L)	5.74 [4.33, 7.98]	6.21 [5.28, 8.08]	***0.012***	5.53 [4.30, 7.85]	6.18 [5.27, 7.90]	***0.009***
HGB (g/L)	132.00 [122.00, 139.00]	131.00 [120.00, 140.00]	0.515	131.00 [121.50, 137.00]	131.00 [119.50 142.50]	0.673
PLT (× 10^9^/L)	216.00 [161.00, 267.00]	216.00 [152.00, 263.00]	0.646	216.00 [165.00, 263.5]	220.00 [153.50 269.00]	0.965
IgG (g/L) (Normal range: 7–17)	11.46 [10.06, 13.86]	11.33 [9.50, 13.26]	0.493	11.46 [9.94, 14.04]	11.53 [9.55, 13.30]	0.701
IgM (g/L) (Normal range: 0.4–2.3)	1.27 [0.91, 1.80]	1.20 [0.74, 1.64]	0.110	1.30 [0.96, 1.80]	1.19 [0.72, 1.60]	***0.049***
IgA (g/L) (Normal range: 0.7–4)	1.91 [1.57, 2.53]	2.12 [1.57, 2.88]	0.095	1.97 [1.57, 2.52]	2.13 [1.62, 2.97]	0.086
Antibody titers						
aCL						
IgG (GPLU/ml) (Normal range: 0–8)	3.89 [2.06, 27.20]	3.49 [1.77, 34.50]	0.756	3.60 [2.06, 28.00]	3.99 [1.78, 33.3]	0.753
IgM (MPLU/ml) (Normal range: 0–8)	3.98 [2.00, 8.95]	3.80 [2.00, 8.85]	0.577	4.12 [2.00, 8.82]	3.71 [0.00, 8.20]	0.877
IgA (APLU/ml) (Normal range: 0–8)	2.50 [2.50, 3.69]	2.50 [2.50, 8.85]	***0.000***	2.50 [2.50, 3.36]	0.00 [0.00, 8.96]	***0.000***
aβ2GP1 (AU/ml)						
IgG (Normal range: 0–16)	4.35 [2.60, 40.80]	3.90 [2.14, 50.20]	0.667	4.18 [2.51, 34.80]	4.09 [2.14 45.15]	0.795
IgM (Normal range: 0–16)	7.23 [2.00, 27.00]	5.46 [2.00, 26.60]	0.716	7.38 [2.00, 26.9]	3.52 [0.00 23.55]	0.870
IgA (Normal range: 0–16)	2.50 [2.50, 5.45]	3.19 [2.50, 12.40]	***0.040***	2.50 [2.50, 4.94]	2.95 [0.00 14.00]	***0.045***
LA	1.20 [1.04, 1.53]	1.13 [1.01, 1.41]	0.222	1.13 [1.02, 1.46]	1.12 [1.12 1.40]	0.578

*p* value lower than 0.05 is presented in bolditalics

### Self-comparison of APS patients prior to and post-SARS-CoV-19 infection

Self-comparisons of clinical manifestations and laboratory indices in 97 APS patients before and after SARS-CoV-2 infection were analyzed (Table 4). There was no change in the incidence of venous thrombosis before and after infection. The incidence of arterial thrombosis increased from 18 cases (18.6%) before infection to 21 cases (21.6%) after infection (*p* < 0.001). The incidence of thrombocytopenia increased from 29 cases (29.9%) before infection to 35 cases (36.1%) after infection (*p* = 0.001). The median WBC count had a significant decrease from 6.20 to 5.53 × 10^9^/L after infection (*p* = 0.117). The median IgG level increased slightly from 11.40 g/L before infection to 11.46 g/L after infection (*p* = 0.036). As for the antibody titer, aCL-IgA had a significant increase (*p* < 0.001), while the median value remained at 2.50 as it is the lowest testing threshold. The median aβ2GPI-IgG level increased slightly from 4.14 to 4.18 (*p* = 0.019). The median aβ2GPI-IgM titer decreased from 9.85 to 7.38 (*p* < 0.001).

**Table 4:** Effect of COVID-19 outbreak on APS patients

	Before COVID-19 infection (*n* = 97)	After COVID-19 infection (*n* = 97)	*p* value
Clinical manifestations			
Venous thrombosis, *n* (%)	32 (33.0%)	32 (33.0%)	1.000
Arterial thrombosis, *n* (%)	18 (18.6%)	21 (21.6%)	***0.000***
Thrombocytopenia, *n* (%)	29 (29.9%)	35 (36.1%)	***0.001***
Laboratory indexes			
WBC (× 10^9^/L)	6.20 [4.47, 7.55]	5.53 [4.30, 7.85]	0.117
HGB (g/L)	132.00 [120.00, 141.00]	131.00 [121.50, 137.00]	0.072
PLT (× 10^9^/L)	215.00 [157.00, 263.00]	216.00 [165.00, 263.50]	0.716
IgG (g/L) (Normal range: 7–17)	11.40 [9.68, 13.69]	11.46 [9.94, 14.04]	***0.036***
IgM (g/L) (Normal range: 0.4–2.3)	1.3 [0.88, 1.78]	1.30 [0.96, 1.80]	0.299
IgA (g/L) (Normal range: 0.7–4)	1.89 [1.44, 2.49]	1.97 [1.57, 2.52]	0.681
Antibody titers			
aCL			
IgG (GPLU/ml) (Normal range: 0–8)	4.45 [2.16, 29.50]	3.60 [2.06, 28.00]	0.057
IgM (MPLU/ml) (Normal range: 0–8)	4.04 [2.00, 8.78]	4.12 [2.00, 8.82]	0.634
IgA (APLU/ml) (Normal range: 0–8)	2.50 [2.50, 3.41]	2.50 [2.50, 3.36]	***0.000***
aβ2GPI (AU/ml)			
IgG (Normal range: 0–16)	4.14 [2.16, 25.85]	4.18 [2.51, 34.80]	***0.019***
IgM (Normal range: 0–16)	9.85 [2.00, 36.20]	7.38 [2.00, 26.90]	***0.000***
IgA (Normal range: 0–16)	2.50 [2.50, 4.60]	2.50 [2.50, 4.94]	0.180
LA	1.16 [1.03, 1.44]	1.13 [1.02, 1.46]	0.926

*p* value lower than 0.05 is presented in bolditalics

### Changes in serum antibodies prior to and post-SARS-CoV-19 infection

**Fig. 2:**
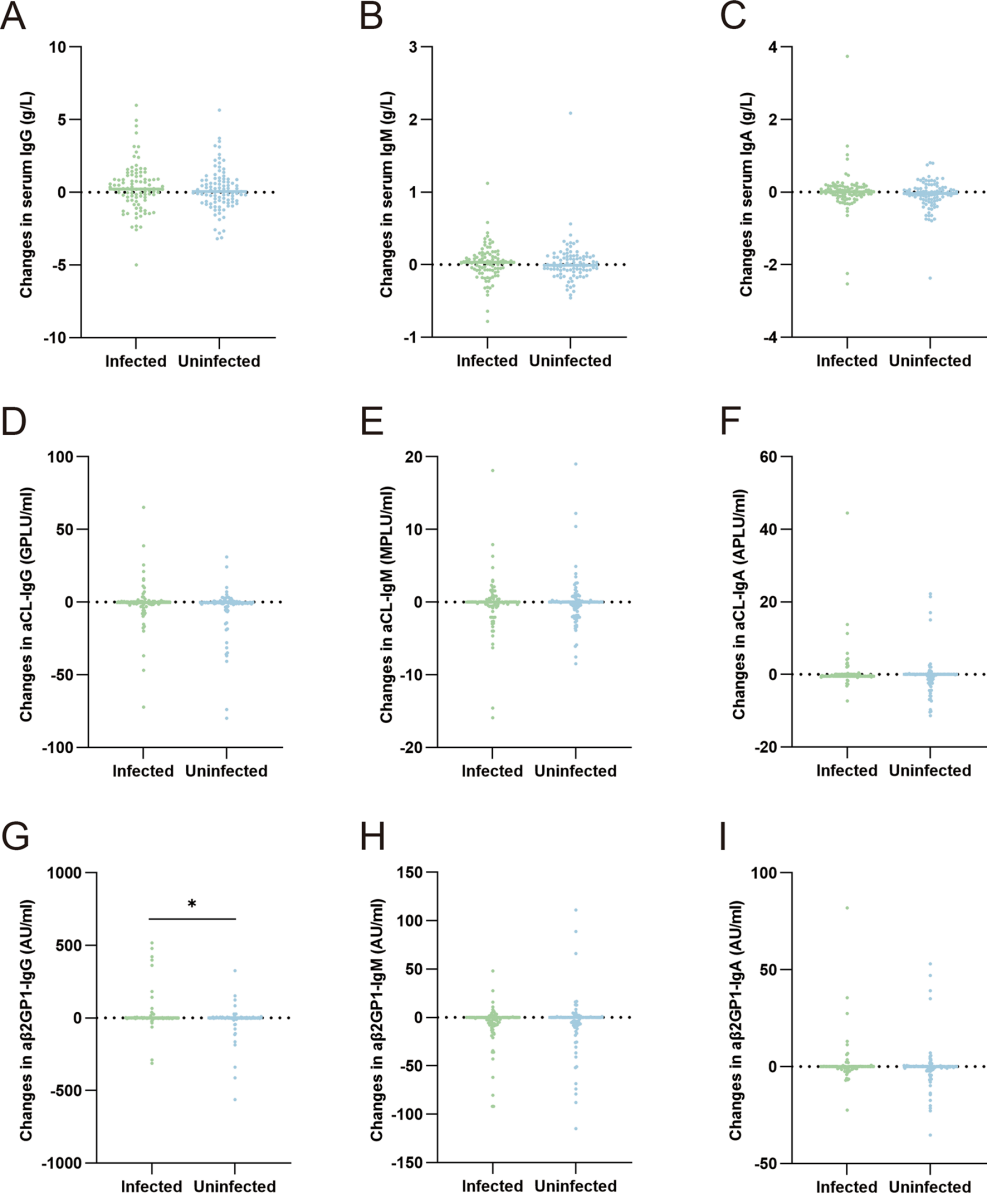
Distribution of changes of serum antibodies. Distributions of changes in serum **A** IgG; **B** IgM; **C** IgA; **D** aCL-IgG; **E** aCL-IgM; **F** aCL-IgA; **G** aβ2GP1-IgG; **H** aβ2GP1-IgM; **I** aβ2GP1-IgA were plotted. *p < 0.05

As changes in serum antibodies could often be observed in the natural course of APS and would not necessarily be associated with disease activity, we then sought to examine whether the changes in serum antibody titer were attributed to SARS-CoV-2 infection. To this end, the changes of serum antibodies were calculated as post-COVID-19 level subtracts pre-COVID-19 level and the 2 groups were compared (Fig. 2). In this set of analyses, only the changes in serum aβ2GP1-IgG showed statistical significance between the 2 groups, with that of the infected group being significantly higher (*p* = 0.031). The majority of the changes were evenly distributed positively and negatively, and no apparent trend of antibody change could be observed from the distribution plot (Fig. 2).

### Platelet decrease after SARS-CoV-2 infection

During the acute phase of SARS-CoV-2 infection, 7 patients presented either newly-onset thrombocytopenia or further decreased platelet count (Table 5). We reviewed the medical status and found these patients had maintained stable platelet count before SARS-CoV-2 infection for at least 3 months, and five of them had mild thrombocytopenia (over 50 × 10^9^/L). All seven patients were treated with either low-dose glucocorticoid, stable DMARDs, or anticoagulant therapy. However, during acute viral infection (within 14 days), these patients all presented a decline in platelet account. With timely adjusted treatment, the platelet can be effectively increased.

**Table 5:** Thrombocytopenia in APS patients after exposing to acute COVID-19 infection

Patient	Age	Sex	Primary/Secondary APS	Baseline treatment	Baseline PLT	Time of PLT decreption	PLT after infection	Treatment	PLT after treatment
1	30	Female	SLE-APS	HCQ 0.2 bid LMWH 4000IU q12h Aspirin 0.1 qd	109	10 days after infection	99	HCQ 0.2 bid LMWH 6000IU q12h Aspirin 0.1 qd AZA 100mg qd	119
2	22	Male	PAPS	TAC 1mg qd Warfarin 3.75 qd HCQ 0.2 bid	113	3 days after infection	17	IVIG 10g qd × 3 days TAC 1mg qd Warfarin 3.75 qd HCQ 0.2 bid	132
3	27	male	SLE-APS	Pre 6.25mg qd TAC 1mg bid HCQ 0.3 qd Aspirin 100mg qd	48	2 days after infection	2	IVIG 20g qd × 3 days Pre 50mg qd TAC 1mg bid HCQ 0.3 qd Aspirin 100mg qd	104
4	43	Female	SLE-APS	TAC 1mg bid Aspirin 75mg qd	100	14 days after infection	54	TAC 1mg bid Aspirin 75mg qd	91
5	55	Female	PAPS	TAC 1mg tid HCQ 0.2 bid	10	3 days after infection	2	IVIG 20g qd × 5 days TAC 1mg tid HCQ 0.2 bid	48
6	68	Male	PAPS	Sirolimus 1mg bid Pre 5mg qod Aspirin 100mg qd	90	4 days after infection	10	IVIG 10g qd × 3 days Sirolimus 1mg bid Pre 5mg qod Aspirin 100mg qd	154
7	54	Female	PAPS	TAC 1mg tid Pre 7.5mg qd Aspirin 50mg qd	65	2 days after infection	38	TAC 1mg tid Pre 7.5mg qd HCQ 0.2 bid	42

## Discussion

In this study, a retrospective analysis was conducted on patients with APS during a concentrated outbreak of acute SARS-CoV-2 infection from October to December 2022. We explored various aspects of clinical manifestations through comparisons made before and after infection and made comparisons in between infected and uninfected APS individuals. Although statistical analyses demonstrated significant increases of the occurrence of venous thrombotic events and aβ2GP1-IgG, the increases were slight and did not necessarily hold clinical significance. Our findings, in general, indicate that acute SARS-CoV-2 infection did not substantially increase the thrombotic events in APS, nor did it lead to an elevation in overall antibody titers over a short timeframe. Specific individuals had a severe decrease in platelet count, suggesting that acute viral infection might somewhat contribute to the exacerbation of thrombocytopenic events.

In our analysis of clinical symptoms in patients with APS following viral infection, we noted relatively high occurrences and severity of symptoms such as high fever, shivering, headache, cough, pharyngalgia, and chest discomfort. In previous meta-analyses of otherwise healthy Chinese individuals infected with SARS-CoV-2, the occurrence of these symptoms was not as high [25, 26]. However, it is critical to consider potential bias because these studies had different virus variants epidemics. Besides, it was hard to match the APS cohort with the population under the circumstances of universal infection. Considering the use of immunosuppressants in some APS patients, frequent and severe symptoms in respiratory tract infection could be reasonable.

Clinical symptoms and APS phenotypes were not significantly different before and after acute viral infection. SARS-CoV-2 infection did not induce a large number of additional thrombotic events as we concerned. Several previous studies have described the presentation of viral infections in APS. In a cross-sectional study, when comparing with unselected APS patients in another study, those with HCV and/or HIV infection present more cardiovascular events and avascular bone necrosis but less deep vein thrombosis. However, the study only presents a combination of chronic viral infections and APS but does not observe the impact of the virus in the APS cohort [27]. Other studies show the prevalence of thromboembolic events in aPL-positive patients with HCV and HBV infection [11]. However, this cannot exhibit the direct relationship between viral infections with APS thrombosis. Other studies report newly occurrence of thrombotic events following viral infections including SARS-CoV-2 and showing that the incidence of thromboembolic complications in patients with SARS-CoV-2 was 35–45%; elevated D-dimer was observed in approximately half of SARS-CoV-2 infected patients [28–32]. However, based on our observations, it is premature to draw conclusions about whether viral infection induces fluctuations in the clinical course of APS. Future investigations should conduct observations over a longer time scale.

As early as the beginning of this century, researchers have noticed that infections may induce aPL production without clinical features necessarily [18]. Multiple studies have found that viral infection induces serum aPL in individuals without APS [11, 33]. The rate of aPL-positive patients in different SARS-CoV-2-infected cohorts is between 24 and 57% [14]. In addition, a systematic review of APS following infection demonstrated that after infections, 55.6% of which were viral, 293 cases developed transient aPLs with or without thromboembolic events; 24.6% of these met the criteria for definitive APS [12]. However, study on the effect of infections on antibody positivity in APS patients is still controversial. The APSANTICO study which included 82 patients with APS demonstrated no increase in general aPL levels, but significant decreases in aCL-IgG and aβ2GP1-IgG antibodies [34]. In the present study, fluctuation of antibodies including aCL-IgA, aβ2GP1-IgG and aβ2GP1-IgM. However, after we reanalyzed the distribution of changes of antibodies and made comparison between the 2 subgroups, only the changes of aβ2GP1-IgG remained significant. Despite the statistical significance, the clinical relevance of this finding may be limited as the median values for all the aforementioned tests remained within negative ranges; additionally, the positivity rates of these antibodies did not differ between before and after SARS-CoV-2 infection. Additional analyses assessing the possibility of antibody class-switch found no corresponding elevation in any other antibody classes. The mechanism underlying this manifestation is currently unknown, and further validation is necessary using a larger cohort. Our current findings suggest that acute viral infection may not significantly induce the elevation of aPL in the short term; nevertheless, strict surveillance of aPL and regular follow-up is still important, as they provide crucial insights into the current status of APS and guide the development of treatment plans.

Regarding the observed platelet decrease, seven patients experienced new-onset thrombocytopenia or had a platelet decrease of no less than 10 × 10^9^/L in a thrombocytopenic background. In a previous work including 51 patients without autoimmune background in our inpatients, none developed thrombocytopenia requiring treatment [35]. These findings align with previous research indicating that various infectious diseases, such as hepatitis C virus, human immunodeficiency virus, *Helicobacter pylori*, and SARS-CoV, can induce thrombocytopenia [36, 37]. We speculate that thrombocytopenia may be caused by two mechanisms. Viral infection may cause thrombocytopenia either by directly interacting with angiotensin-converting enzyme 2 (ACE2) or by coagulation and inflammation activation [38, 39]. Dysregulation in the immune system of patients with APS during infection may also play a vital role [39, 40]. As the decline in platelet counts in these patients mostly occurred within one week after infection, with timely adjustments to treatment, most of the decreases could be reversed. These results indicate that monitoring platelet levels in patients with APS after SARS-CoV-2 infection is crucial.

The present study has certain limitations. The sample size was relatively small, and the study was conducted at a single clinical center. Additionally, since all infections occurred after November 2022, the duration of postinfection follow-ups was limited. A more extended observation period could potentially reveal additional effects of SARS-CoV-2 infection in patients with APS. We briefly examined the impact of SARS-CoV-2 vaccination on infection rates and disease severity. However, the vaccine doses and manufacturers were not standardized, introducing potential bias. Future investigations with larger cohorts and extended follow-up periods are warranted to comprehensively assess the long-term relationships between SARS-CoV-2 and APS.

## Conclusions

In our retrospective analysis, acute SARS-CoV-2 infection did not substantially increase the occurrence of thrombotic events, nor did it lead to an elevation in antibody titers over a short timeframe. The SARS-CoV-2 infection may cause mild fluctuations in some APS antibodies, but these changes had no clinical significance. Clinical APS symptoms and phenotypes were not significantly changed before and after acute viral infection.

## Data Availability

All relevant data are included within the manuscript. Clinical data of specific participants are available from the corresponding author on reasonable request.
